# Enhanced Deficits in Long-Term Potentiation in the Adult Dentate Gyrus with 2^nd^ Trimester Ethanol Consumption

**DOI:** 10.1371/journal.pone.0051344

**Published:** 2012-12-05

**Authors:** Jennifer L. Helfer, Emily R. White, Brian R. Christie

**Affiliations:** 1 Division of Medical Sciences, University of Victoria, Victoria, British Columbia, Canada; 2 Department of Biology, University of Victoria, Victoria, British Columbia, Canada; 3 Graduate Program in Neuroscience, University of Victoria, Victoria, British Columbia, Canada; 4 Graduate Program in Neuroscience and the Department of Cellular and Physiological Sciences, University of British Columbia, Vancouver, British Columbia, Canada; 5 The Brain Research Centre, University of British Columbia, Vancouver, British Columbia, Canada; University of Nebraska Medical Center, United States of America

## Abstract

Ethanol exposure during pregnancy can cause structural and functional changes in the brain that can impair cognitive capacity. The hippocampal formation, an area of the brain strongly linked with learning and memory, is particularly vulnerable to the teratogenic effects of ethanol. In the present experiments we sought to determine if the functional effects of developmental ethanol exposure could be linked to ethanol exposure during any single trimester-equivalent. Ethanol exposure during the 1^st^ or 3^rd^ trimester-equivalent produced only minor changes in synaptic plasticity in adult offspring. In contrast, ethanol exposure during the 2^nd^ trimester equivalent resulted in a pronounced decrease in long-term potentiation, indicating that the timing of exposure influences the severity of the deficit. Together, the results from these experiments demonstrate long-lasting alterations in synaptic plasticity as the result of developmental ethanol exposure and dependent on the timing of exposure. Furthermore, these results allude to neural circuit malfunction within the hippocampal formation, perhaps relating to the learning and memory deficits observed in individuals with fetal alcohol spectrum disorders.

## Introduction

When ethanol is consumed during pregnancy it distributed throughout the body of both the mother and fetus. Because ethanol is both lipid and water soluble, it freely passes through the placental membrane and infiltrates all tissues and organs of the fetus. This can result in a disruption of normal development and can set the stage for a myriad of lifelong struggles. Although many advances have been made in our understanding of the detrimental effects ethanol has on a developing fetus, ethanol consumption during pregnancy is fairly common. In a recent survey of women in the United States of America, 30.3% reported drinking at some point during their pregnancy. Even more alarming, 8.3% of these women reported binge drinking; binge drinking was defined as four or more drinks on any one occasion [1].

Out of every 100 births, an estimated two to five children are born each day with an ethanol related disorder [2]. Disorders resulting from developmental ethanol exposure are classified under the umbrella term fetal alcohol spectrum disorders (FASDs). These disorders include fetal alcohol syndrome, partial fetal alcohol syndrome, alcohol related birth defects, and alcohol related neurodevelopment disorder [3]. Disorders within the spectrum are characterized with some or all of the following problems: facial dysmorphology, growth deficits, physical abnormalities, and/or central nervous system damage. Furthermore, these unfortunate individuals will suffer their entire lives as these disabilities only get worse with age [4].

The exact causal factor for why a spectrum of adverse effects results from developmental ethanol exposure is unknown; it may be that there is no single source. We are aware of a number of risk factors that can be linked to particular clinical diagnosis within the spectrum of ethanol related disorders, including the dose and developmental timing of exposure [5], [6]. Over the nine months of human development each structure and organ in the fetal body develops at its own rate. During this time, development of these structures/organs can be broken up into stages and critical periods. The characteristic facial features of FAS are associated with high ethanol consumption during the sixth to ninth week of gestation (1^st^ Trimester) [7]. Whereas, the resulting behavioral and cognitive deficits depend on the timing of their associated neural systems development but generally occur from consumption during the second and third trimester [8]. However, there is a lack of information in regards to the timing of ethanol exposure and the teratogenic effects on dentate gyrus (DG) synaptic plasticity. Previous reports have only examined the effects of exposure during the 1^st^ and 2^nd^ trimester-equivalents [9], [10], [11], [12].

When studying the effects of ethanol on the development of the hippocampal formation in rodent models it is important to control for the difference in the time course of development. Not only is human in utero development much longer than that of a rodent (nine months vs. 22 days), the hippocampal formation also has different developmental properties. In humans development of the hippocampal formation begins during the second half of the 2^nd^ trimester and continues throughout the 3^rd^ trimester [13]. In rodents, hippocampal development begins during the second half of the gestational period and continues following birth to around postnatal day (PD) 14 [14], [15]. Thus, gestational days (GD) 1–10 in rodents (mouse and rat specific) are thought to be equivalent to the 1^st^ trimester of human pregnancy in terms of neuronal development, GD 11–22 equivalent to the 2^nd^ trimester, and PD 1–14 equivalent to the 3^rd^ trimester [16], [17]. Typically most rodent studies either give ethanol during the prenatal period (GD 1–22) of development or during the postnatal period (GD 4–9) to mimic either a 1^st^ and 2^nd^ trimester exposure or a 3^rd^ trimester exposure, respectively.

**Figure 1:**
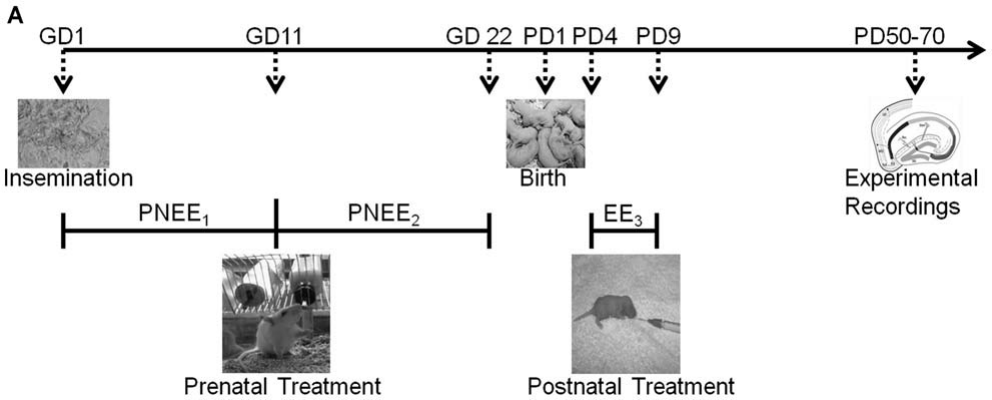
Generation of Animal Model. The presence of sperm was used to indicate gestation day 1 (GD 1). Rat dams or pups were exposed to a liquid diet containing ethanol either during the: (i) 1st trimester equivalent  =  prenatal ethanol exposure 1 (PNEE_1_); (ii) 2^nd^ trimester equivalent  =  prenatal ethanol exposure 2 (PNEE_2_); or (iii) 3^rd^ trimester equivalent  =  ethanol exposure 3 (EE_3_). Appropriate pair-fed and ad libitum animals were also reared. Dentate gyrus field recordings were conducted in early adulthood (postnatal day (PD) 50–70).

The present study established critical periods of rat DG development in relation to developmental ethanol exposure (1^st^, 2^nd^, or 3^rd^ trimester-equivalent) induced alterations in synaptic plasticity. We examined whether the administration of ethanol during a single trimester-equivalent could influence the severity of altered dentate gyrus function in adulthood. In an effort to determine periods of vulnerability within DG development, we exposed pregnant dams or rat pups to ethanol during each individual trimester and evaluated the long-term effects of ethanol exposure on synaptic plasticity in the adult DG (see Figure 1 for timeline). In separate groups, we also examined how restricted nutritional intake in each trimester can impact synaptic plasticity. This manipulation is often performed as a “control group” in most studies, however our data indicate that this is in fact a separate experimental group.

## Results

### Maternal and Offspring Parameters

As is indicated in Table 1, all pregnant dams continued to gain body weight throughout gestation. There were no significant differences in percent weight gain over gestation (F(4,13)  = 38.28, p = 0.083), in gestation length (F(4,13)  = 0.97, p = 0.455), or in litter size (F(4,16)  = 1.06, p = 0.408) among ad libitum (AL), prenatal ethanol exposure during the 1^st^ trimester equivalent (PNEE_1_), and prenatal ethanol exposure during the 2^nd^ trimester equivalent (PNEE_2_) dams.

**Table 1:** Maternal Parameters.

Treatment Group	% Weight Gain	Length of Gestation (days)	Litter Size (pup #)	BEC (mg/dl)
AL	27.60±3.91	22.40±0.24	14.25±1.42	na
PF_1_	35.02±0.73	21.67±0.33	13.67±1.45	na
PNEE_1_	39.20±3.40	21.75±0.25	15.00±1.29	155.20±9.61
PF_2_	28.00±1.26	22.00±1.00	16.50±1.50	Na
PNEE_2_	28.84±1.96	22.00±0.00	17.75±1.11	142.47±8.36
EE_3_ Male*	na	na	na	347.40±21.30
EE_3_ Female*	na	na	na	314.70±15.85

The values represent mean ± SEM. AL, ad libitum; BEC, blood ethanol concentration; EE, ethanol exposed; na, not applicable; PF, pair-fed; PNEE, prenatal ethanol exposed.

* BEC values taken from pups on postnatal day 4.

Similar blood ethanol concentration (BEC) levels were acquired from PNEE_1_ (155.20±9.61 mg/dl) and PNEE_2_ (142.47±8.36 mg/dl) dams (t(6)  = 0.99, p = 0.356). The average BEC for male offspring exposed to ethanol during the 3^rd^ trimester equivalent (EE_3_) was 347.40±21.29 mg/dl and for EE_3_ female offspring was 314.70±15.85 mg/dl. These levels are similar to those found in previously published studies [18], [19]. Although males had slightly higher BECs than females, this difference did not reach significance (t(16)  = 1.23, p = 0.42). Similar differences in gender BEC levels have been reported elsewhere [20].

Offspring were weighed throughout the postnatal period until their experimental use in adulthood. Following birth, weights taken on the litter cull date (PD 2/3) were significantly affected by developmental treatment (F(6,103)  = 12.44, p<0.001) and gender (F(1,103)  = 13.62, p<0.001). Furthermore, no significant interaction between developmental treatment and gender was found. At this early age, female pups weighed significantly less than males (p<0.001). All prenatally treated pups' weights were significantly reduced compared to AL (PNEE_1_ vs. AL: p = 0.007; pair-fed during the 1^st^ trimester equivalent (PF_1_) vs. AL: p<0.001; PNEE_2_ vs. AL: p = 0.006; pair-fed during the 2^nd^ trimester equivalent (PF_2_) vs. AL: p = 0.029). Considering EE_3_ and sham-intubated (SI_3_) pups at this time had not received developmental treatment, their weights were not different from AL and were significantly higher than prenatal treated pups (p<0.001). Weights were again taken on PD 8 from AL, EE_3_, and SI_3_ pups to test whether postnatal treatment affected weight. At this time point, there was a main effect of postnatal treatment (F(2,49)  = 5.45, p = 0.007), revealing that EE_3_ offspring weighed significantly less than AL (p = 0.016) and SI_3_ (p = 0.012) offspring. There was no main effect of gender and no interaction between the two variables.

At the time of electrophysiological experimentation (PD 50–70), there was a main effect of developmental treatment (F(6,104)  = 3.92, p = 0.001), gender (F(1,104)  = 313.60, p<0.001), and a significant interaction between treatment and gender (F(6,104)  = 3.62, p = 0.002) on weight. All female offspring weighed significantly less then male offspring (p<0.001), a common gender difference at this age [21]. Upon assessment of the interaction, PNEE_1_ and PNEE_2_ males weighted significantly less than AL males (PNEE_1_ vs. AL: p = 0.030; PNEE_2_ vs. AL: p<0.025). Furthermore, PNEE_1_ males weighed significantly less than PF_1_ males (p = 0.001). However, weights did not differ between the three ethanol treated groups (Table 2).

**Table 2:** Offspring Developmental Data.

Gender	Treatment Group	PD 2/3a, b	PD 8^c^	PD 50–70^a^
Male	AL (n = 14)	8.96±0.20	20.31±1.05	415.71±10.06
	PF_1_ (n = 6)	7.59±0.06	na	447.30±21.79
	PNEE_1_ (n = 10)	7.82±0.10	na	357.30±15.79 d,e
	PF_2_ (n = 7)	8.00±0.18	na	366.57±27.46
	PNEE_2_ (n = 8)	7.63±0.22	na	352.25±13.41^d^
	SI_3_ (n = 10)	8.98±0.36	20.22±0.74	418.71±17.62
	EE_3_ (n = 7)	8.85±0.49	16.82±0.57	373.00±19.90
Female	AL (n = 12)	7.83±0.22	17.95±0.53	243.58±9.96
	PF_1_ (n = 11)	6.86±0.03	na	267.18±7.34
	PNEE_1_ (n = 6)	7.06±0.01	na	251.67±11.72
	PF_2_ (n = 8)	7.31±0.27	na	266.00±10.03
	PNEE_2_ (n = 7)	7.36±0.11	na	256.28±13.52
	SI_3_ (n = 9)	9.01±0.43	17.24±0.60	265.50±12.59
	EE_3_ (n = 6)	8.50±0.59	19.10±0.79	258.89±6.89

The values represent mean weight (g) ± SEM. AL, ad libitum; BEC, blood ethanol concentration; EE, ethanol exposed; PD, postnatal day; PF, pair-fed; PNEE, prenatal ethanol exposed; SI, sham intubated.

a Males weighed more than females; *p*<0.01.

b Prenatal treatment groups (PF_1_, PNEE1, PF_2_, and PNEE_2_) weighed less than both AL and postnatal treatment (SI_3_ and EE_3_) groups; *p*<0.05.

c EE_3_weighed less than both AL and SI_3_ groups; *p*<0.05.

d Weighed less than AL group; *p*<0.05.

e Weighed less than PF_1_ group; *p*<0.01.

### Input/Output and Paired Pulse Plasticity

**Figure 2:**
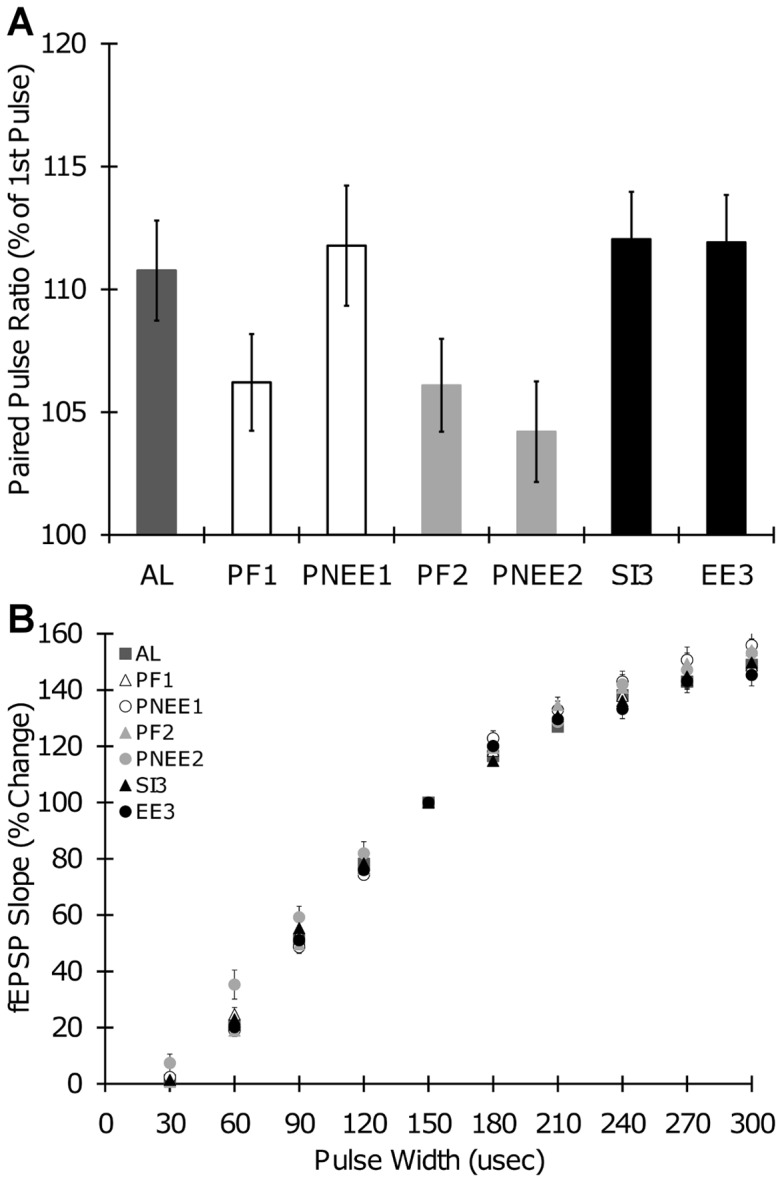
Input/Output Curves and Paired Pulse Ratios. (A) Paired pulse (PP) ratios with a 50 ms inter-stimulus-interval. (B) Input/output (I/O) curves showing fEPSP slope plotted against stimulus strength for all developmental treatment groups. Baseline synaptic strength increased with increased stimulation in all slices. AL, ad labium; PNEE_1_, prenatal ethanol exposure 1; PNEE_2_, prenatal ethanol exposure 2; EE_3_, ethanol exposure 3; PF_1_, pair-fed 1; PF_2_, pair-fed 2; SI_3_, sham intubated 3.

To determine whether prenatal ethanol exposure during any given trimester-equivalent alters neural transmission, we conducted two experiments. First, paired pulse (PP) plasticity was examined to determine whether ethanol exposure altered presynaptic transmitter release. PP ratios were significantly different among treatment groups (F(6,297)  = 2.76, p = 0.012), however post-hoc analysis did not reveal any significant differences (PNEE_1_: 111.76±2.45; PNEE_2_: 104.20±2.05; EE_3_: 111.91±1.93; PF_1_: 106.21±1.96; PF_2_: 106.10±1.89; SI_3_: 112.04±1.93; AL: 110.76±2.04) (Figure 2A). Therefore, developmental ethanol exposure and pair-feeding/sham-intubation had no effect on PP ratios when induced with a 50 ms inter-pulse-interval. There was no effect of gender on PP ratios (F(1,297)  = 0.49, p = 0.484). Second, excitatory synaptic transmission was characterized through input/output (I/O) function. In all slices, the slope of the fEPSP significantly increased with increasing stimulation (repeated measures ANOVA; F(8,2360)  = 3255.77, p<0.001). Developmental treatment had no significant effect on I/O function (F(6,295)  = 1.53, p = 0.169), regardless of gender (F(1,295)  = 0.64, p = 0.424) (Figure 2B).

### Long-term Potentiation

**Figure 3:**
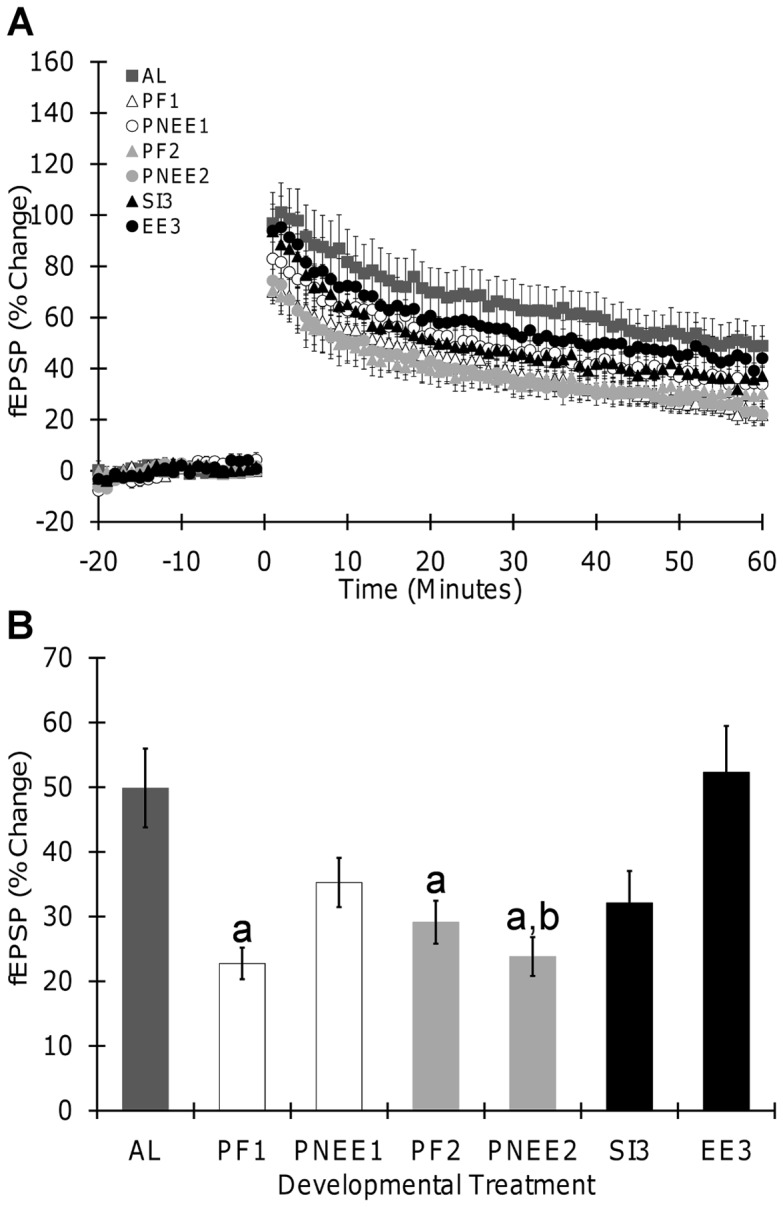
Long term-potentiation induced by theta burst stimulation in hippocampal slices. (A) Field excitatory postsynaptic potential (fEPSP, slope) recorded before and after TBS in slices from all treatment groups. (B) Effects of developmental ethanol exposure on LTP. No significant main effect of sex was obtained, thus data from male and female offspring were pooled together. Each point and bar graph shown is the mean ± SEM. Insert illustrates samples of traces obtained from corresponding groups; fEPSP recorded before (gray) or 1 hour (black) after conditioning stimulation are superimposed. Scale bar represents 0.5 mV by 10 ms. a, significantly different from AL; b, significantly different from EE_3._ AL, ad libitum; PNEE_1_, prenatal ethanol exposure 1; PNEE_2_, prenatal ethanol exposure 2; EE_3_, ethanol exposure 3; PF_1_, pair-fed 1; PF_2_, pair-fed 2; SI_3_, sham intubated 3.

To test whether any one trimester is more vulnerable to the long-term effects of developmental ethanol exposure, long-term potentiation (LTP) was compared across trimester equivalents. Both theta-burst stimulation (TBS) and high-frequency stimulation (HFS) were used to elicit LTP. Both TBS and HFS produced significant LTP in all offspring. LTP data are summarized in Table 3 and graphed in Figure 3 (TBS) and 4 (HFS).

**Table 3:** Potentiation in Hippocampal Slices.

Gender	Treatment Group	Conditioning Stimulus	LTP
Male	AL	TBS (n = 10)	59.55±10.38
		HFS (n = 10)	51.27±5.38
	PF_1_	TBS (n = 8)	23.13±4.67
		HFS (n = 9)	48.72±8.18
	PNEE_1_	TBS (n = 13)	36.57±4.96
		HFS (n = 15)	55.38±6.84
	PF_2_	TBS (n = 10)	31.94±5.40
		HFS (n = 16)	39.93±3.90
	PNEE_2_	TBS (n = 10)	27.48±3.59
		HFS (n = 10)	27.14±7.61
	SI_3_	TBS (n = 10)	25.83±5.90
		HFS (n = 12)	61.32±6.13
	EE_3_	TBS (n = 13)	47.90±7.39
		HFS (n = 14)	58.25±6.12
Female	AL	TBS (n = 10)	40.20±5.28
		HFS (n = 10)	54.18±4.64
	PF_1_	TBS (n = 13)	22.53±2.88
		HFS (n = 14)	38.64±6.19
	PNEE_1_	TBS (n = 6)	32.41±5.98
		HFS (n = 11)	40.66±6.23
	PF_2_	TBS (n = 11)	26.57±4.10
		HFS (n = 14)	40.28±6.27
	PNEE_2_	TBS (n = 12)	20.79±4.57
		HFS (n = 11)	32.85±10.08
	SI_3_	TBS (n = 9)	39.08±7.80
		HFS (n = 9)	67.00±13.33
	EE_3_	TBS (n = 9)	58.64±14.21
		HFS (n = 12)	78.41±5.37

The values represent mean fEPSP slope (% change) ± SEM. n corresponds to the number of slices. AL, ad libitum; BEC, blood ethanol concentration; EE, ethanol exposed; HFS, high frequency stimulation; PF, pair-fed; PNEE, prenatal ethanol exposed; SI, sham intubated; TBS, theta burst stimulation.

Following TBS, a significant main effect of developmental treatment (F(6,130)  = 6.13, p<0.001) revealed that LTP magnitude was lower in PF_1_(p<0.001), PF_2_ (p = 0.029), and PNEE_2_ (p<0.001) offspring when compared to AL. LTP was attenuated by 52% in PNEE_2_, 54% in PF_1_, and 41% in PF_2_ offspring as compared to AL offspring (Figure 3B). Comparisons of ethanol treatment across the different trimesters demonstrated trimester specific effects. Specifically, the magnitude of LTP in PNEE_2_ offspring was 54% less than that observed in EE_3_ offspring (p<0.001). When comparing the magnitude of LTP in EE_3_,SI_3_,and AL offspring, the only significant difference found was between EE_3_ and SI_3_ offspring (p = 0.039). There was no main effect of gender or any significant interactions between variables.

**Figure 4:**
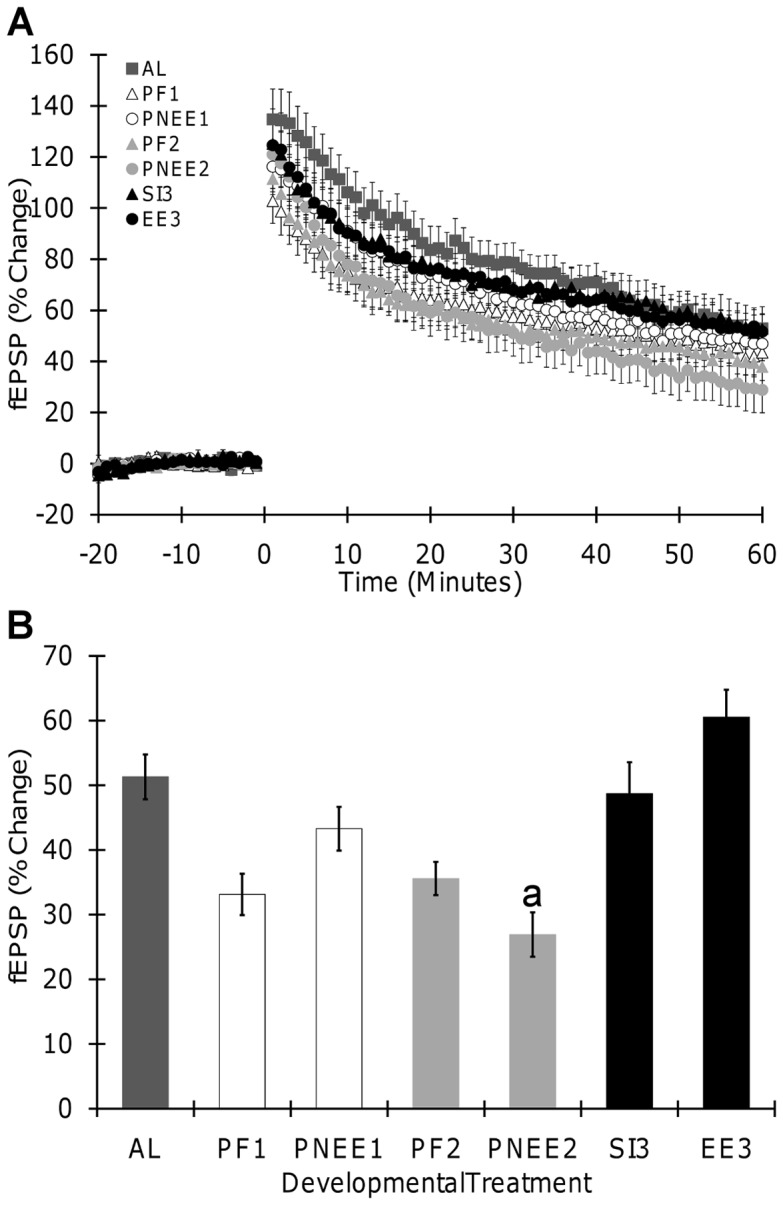
Long term-potentiation induced by high frequency stimulation in hippocampal slices. (A) Field excitatory postsynaptic potential (fEPSP, slope) recorded before and after HFS in slices from all treatment groups. (B) Effects of developmental ethanol exposure on LTP. No significant main effect of sex was obtained, thus data from male and female offspring were pooled together. Each point and bar graph shown is the mean ± SEM. Insert illustrates samples of traces obtained from corresponding groups; fEPSP recorded before (gray) or 1 hour (black) after conditioning stimulation are superimposed. Scale bar represents 0.5 mV by 10 ms. a, significantly different from AL. AL, ad libitum; PNEE_1_, prenatal ethanol exposure 1; PNEE_2_, prenatal ethanol exposure 2; EE_3_, ethanol exposure 3; PF_1_, pair-fed 1; PF_2_, pair-fed 2; SI_3_, sham intubated 3.

Interestingly, slightly different results were obtained from recordings in which LTP was induced by HFS. Following HFS, a significant main effect of developmental treatment (F(6,153)  = 7.47, p<0.001) revealed that LTP magnitude was reduced only in PNEE_2_ (p = 0.037, 43%) offspring when compared to AL (Figure 4B). Comparisons of ethanol treatment across the different trimesters demonstrated trimester specific effects. Specifically, the magnitude of LTP in PNEE_2_ offspring was 55% less than that observed in EE_3_ offspring (p<0.001). There was no main effect of gender or any significant interactions between variables. Although there is a significant reduction in LTP magnitude between PNEE_2_ and AL offspring, the magnitude of LTP did not differ between PNEE_2_ and PF_2_ (p = 0.758) or PF_2_ and AL (p = 0.517), suggesting that nutritional alterations caused by ethanol consumption mediate some of the deficits observed in PNEE2 offspring.

Consistent across the two LTP studies, PNEE_2_ lead to significant attenuation of LTP in the adult DG. This reduction in LTP following ethanol exposure during the 2nd trimester equivalent did not correlate with the reduction in adult weight observed in these animals (r = −0.028, r^2^<0.001, p = 0.631). Together, these results indicate that ethanol exposure during the 2^nd^ trimester equivalent produces the greatest ethanol induced alterations in LTP.

## Discussion

These findings are the first to show trimester specific effects (i.e. individual trimester exposures) of developmental ethanol exposure on DG LTP. Furthermore, this is the first study to examine the effects on developmental ethanol exposure on LTP in the adult DG using in vitro recording techniques. Field recordings revealed that exposure to ethanol restricted to the 2^nd^ trimester equivalent of pregnancy resulted in severe attenuation of LTP. However, ethanol exposure during the 1^st^ or 3^rd^ trimester equivalent did not have a significant effect on LTP. This finding demonstrates that the timing of exposure is a key factor in the teratogenic effects of ethanol and that the DG is particularly vulnerable during the 2^nd^ trimester equivalent.

The effect of PNEE_2_ on DG LTP is similar to those reported previously where exposure lasted throughout the 1^st^ and 2^nd^ trimester equivalents [9], [10], [12]. Interestingly, ethanol exposure restricted to the 1st trimester equivalent did not alter LTP, suggesting that the deficits observed in these earlier studies may be the result of exposure during the 2^nd^ trimester. The decreased adult weight in prenatal ethanol exposed males is similar to that seen in nutrition depravation studies [22], [23]. However other studies have not shown this decrease following prenatal ethanol exposure [9]. The difference in LTP in PNEE_2_ offspring is probably not due to weight reductions since there was no correlation between adult weight and LTP magnitude found. Furthermore, these deficits are not likely to result from long-term deficits in basal neural transmission. The finding of no significant effect of pre or postnatal treatment on I/O function suggests that these developmental insults do not impact basic cellular properties of the dentate granule cell population.

Previous reports have shown that pre and postnatal ethanol exposure have some effect on LTP in the CA1 region of the hippocampal formation. Swartzwelder and colleagues [24] were the first to investigate the effects of developmental ethanol exposure on CA1 LTP, reporting decreased LTP in adult offspring that had been prenatally exposure to ethanol throughout gestation (GD 1–22). Interestingly, exposer to ethanol during the end of the 1^st^ trimester equivalent and throughout the 2^nd^ trimester equivalent (GD 8–20/22) does not produce these long lasting CA1 LTP deficits [25], [26], suggesting that the CA1 and DG are differentially affected by the timing of prenatal ethanol exposure. Similar to the results obtain in the DG, Bellinger and colleagues [27] found that exposure to ethanol during the 3^rd^ trimester equivalent did not have an effect on CA1 LTP in adult offspring. Additionally, work conducted in a guinea pig model of FASD has shown trimester specific effects of developmental ethanol exposure on LTP in the adult CA1 [28], [29]. Exposure to ethanol during all three trimester equivalents (all three trimester equivalents being prenatal in guinea pigs) resulted in decreased CA1 LTP in both male and female offspring [28], whereas exposure during the 3^rd^ trimester equivalent did not result in CA1 LTP deficits [29].

### 2^nd^Trimester Equivalent Ethanol Exposure and Long-Lasting Effects on the DG

The reduction in LTP in the adult DG following exposure to ethanol during the 2^nd^ trimester corresponds with behavior studies where mid-gestational ethanol exposure via a liquid diet (35% ethanol derived calories) was sufficient to impair radial arm maze acquisition in both adolescent (PD 26) and adult (PD 80) rats [30]. Although further studies are needed to further link memory impairments with altered DG LTP in FASD, these findings do help pin point possible developmental processes altered by ethanol neurotoxicity.

The mechanism(s) by which developmental ethanol exposure induces long-lasting behavioral and cognitive deficits is not fully understood. However, our findings suggest that the DG is vulnerable to ethanol during the period of development corresponding to the 2nd trimester of human pregnancy. Thus, by examining the developmental processes taking place during this time, we may be able to narrow down the possible targets of ethanol teratogenic effects.

During the 2^nd^ trimester equivalent, generation of granule cells and interneurons takes place, cells migrate to the future site of the DG, and neuronal networks begin to form. Exposure to ethanol during this stage of development has been reported to alter neuron and glial proliferation, migration, and survival in the cortex, brain stem, and hippocampal CA1 [31], [32], [33], [34]. Furthermore, ethanol exposure during the second trimester equivalent in rodents leads to intrauterine growth retardation and microcephaly [35], [36]. However, limited studies have focused primarily on the DG. Therefore, it is likely that alterations in one or all of these developmental processes lead to long-lasting neurobehavioral disabilities.

Cell proliferation and differentiation along with programmed cell death are important processes in the development of the DG [37]. As early as GD 10, granule cells and their precursors start forming. Peak generation of hippocampal interneurons occurs between GD 13 and 14 in the DG [38], [39]. One of two studies examining the effects of prenatal ethanol exposure on the DG reported no change in the number of dorsal granule cells in adult rats aged 60 that had been exposed to ethanol prenatally between GD 10 and 21 [31]. Similarly in a later study where ethanol exposure included the late 1st trimester and entire 2nd trimester equivalents (GD 6–21), no alteration in the number of DG granule cells was detected in PD 30–35 rats [33].

In addition to cell genesis, ethanol may also affect differentiation and survival of newly generating cells. Ethanol inhibited differentiation of cultured neural stem cells exposed to ethanol (20–100 mM) on GD14 [40]. When neural stem cells were exposed to insulin-like growth factor 1 or brain-derived neurotrophic factor in addition to ethanol, differentiation was no longer affected, suggesting that ethanol alters neural stem cell differentiation through inhibition of neurotrophic factor signaling. Interestingly, neuronal survival was not affected in this study [40]. Developmental ethanol exposure can also alter cell death processes and lead to substantial apoptosis. Through increases in Bax expression and associated processes, exposure to ethanol during the 2^nd^ trimester equivalent in mice promoted increased apoptosis within the developing hippocampal formation [41]. Bax is a Bcl-2 family protein involved in the promotion of apoptosis through initiation of mitochondria release of cytochrome c which in turns stimulates caspase 3,6, 7, and 9 [42]. Other studies have also demonstrated increased apoptosis following developmental ethanol exposure through GABA receptor enhancement and NMDA receptor blockade [43].

Beginning around GD 14, granule precursor cells start migrating from the dentate neuroepithelium and form the secondary dentate matrix [44], [45], [46]. Proliferating cells within the secondary matrix further migrate, forming the tertiary dentate matrix and parts of the granule cell layer of the DG, which starts to become noticeable right before birth [14], [47]. Abnormalities in neuronal and glial migration have been reported in the cortex, brainstem and cerebellum following prenatal ethanol exposure [48], [49], [50], [51]. It will be of importance for future studies to examine the effects of developmental ethanol exposure on migration of DG cells and the formation of the proliferative matrixes, as alterations may contribute to the long-term effects of developmental ethanol exposure.

Synaptic fibers from the entorhinal cortex first appear in the DG around GD 18 [52], [53]. However the number of synapses formed does not reach adult levels until around PD 25 [54]. Any alteration in the entorhinal cortex axonal targeting into the DG may result in reduced synaptic connections or a decrease in the number of fibers that may make it more difficult to evoke long-lasting potentiation. McNaughton, Douglas, and Goddard [55] demonstrated that long-lasting enhancement of perforant path synapses requires the coactivity of a large number of perforant path fibers. Interestingly, West and Hamre [56] showed that prenatal ethanol exposure during GD 11–21 had no detectable effect on granule cell axon branching, indicating that at least hippocampal mossy fiber development in rats (i.e. axons) is not altered.

Ethanol exposure during the 2^nd^ trimester equivalent may also interfere with the function of ligand-gated ion channels thereby resulting in long-lasting hippocampal damage. Ligand-gated ion channels not only mediate fast excitatory and inhibitory synaptic transmission, but also play an important role in the development of the central nervous system and have been implicated in the pathophysiology of FASD [57]. NMDA receptors are involved in many developmental processes including proliferation, differentiation, migration, and synapse formation. Following exposure to ethanol between GD 16–21, there was a significant reduction in NMDA sensitive 3H-glutamate binding sites in the DG [58], [59]. Of importance, this decrease was not observed following exposure during the 1^st^ or 3^rd^ trimester equivalent [59]. Similarly, Diaz-Granados and colleagues [60] reported that prenatal ethanol exposure between GD 12 to GD 18 resulted in a decrease of ^3^H-MK-801 binding in the hippocampus, further supporting the increased teratogenic effects of ethanol during the 2^nd^ trimester equivalent. Nixon and colleagues [61] showed that the expression of the GluN2A subunit of the NMDA receptor was significantly increased in the hippocampus of PD10 rats following exposure to ethanol throughout the 2^nd^ and 3^rd^ trimester equivalents. Therefore, alterations in the NMDA receptor complex following exposure during the 2^nd^ trimester equivalent may underlie abnormities in hippocampal development associated with FASDs and lead to long-term alterations in hippocampal function, such as those reported in LTP.

### Lack of Gender Specific Effects of Developmental Ethanol Exposure

Observations of the effects of developmental ethanol exposure on hippocampal LTP are typically only done in male offspring, with very few studies reporting the examination of females offspring [11], [28], [29]. In an effort to expand on gender-specific alterations of LTP, we examined the effects of developmental ethanol exposure in both male and female offspring. Throughout the current study gender differences were not observed in any of our measures except for adult weight which is a common gender difference at this age [21]. Therefore, LTP is equally reduced in male and female offspring following ethanol exposure during the 2^nd^ trimester equivalent. Similarly, Richardson and colleagues found that exposure to ethanol thought all three trimesters of gestation reduced CA1 LTP in young adult/late adolescent male and female guinea pigs [28]. However, when DG LTP is observed in adolescent males and females following exposure to ethanol during the 1^st^ and 2^nd^ trimester equivalents, LTP is reduced in males but enhanced in females [11]. Therefore, comparison of our current findings with these suggest that there may be a difference in the ontogeny of developmental ethanol effects on LTP when it comes to gender, one that may relate to sexual maturation [62], [63].

### Nutrition, Fetal Development, and Synaptic Plasticity

During pregnancy, nutritional status of the mother plays an important role in the fetus's development. Both undernutrition and malnutrition interfere with the development of the hippocampal formation [64]. Undernutrition is defined as the availability of all nutrients being required by a species in their diet, but the amount available is insufficient; whereas malnutrition is defined as receiving a diet in which one or more essential nutrients is missing or the wrong proportions [64].

In the current study, we found LTP was negatively affected in our pair-feeding offspring in one of two experimental (TBS, but not HFS). Although the liquid diet given to PF dams provides adequate nutrition, the quantity of food is limited to correspond to that consumed by PNEE dams. Therefore, PF offspring serve as a model of undernutrition and provide a means of distinguishing the effects of ethanol and caloric restriction on development. However, ethanol consumption not only results in the consumption of less food [65], [66], but also impairs nutrient and vitamin absorption [67], [68] and interferes with nutritional supply to the fetus [69]. Thus, even with the use of a PF group, the nutritional effects that accompany ethanol consumption are difficult to separate from the teratogenic effects of ethanol alone [69], [70], [71].

Unlike the time specific alterations observed after developmental ethanol exposure, undernutrition affects development during both the 1st and 2nd trimester equivalent, indicating that undernutrition and ethanol may be affecting development through different mechanisms. These finding are consistent with earlier reports indicating that undernutrition lowers the rate of all brain growth events to the same extent [72]. Furthermore, the reduction of PTP and LTP following prenatal caloric restriction are in agreement with previously reported findings [73]. Nevertheless, this is the first report to examine the effects of caloric restriction within individual trimesters on LTP in the adult male and female DG.

The reduction of LTP following prenatal caloric restriction is in agreement with previously reported findings, further stressing the importance of nutritional status during pregnancy. Large prenatal caloric restriction (50% reduction of control intake) and prenatal protein malnutrition result in the impairment of both the establishment and maintenance of DG-LTP in adult male offspring [73], [74], [75]. There is also experimental evidence indicating that prenatal malnourishment alters excitatory glutamatergic activity that may relate to alterations in LTP observed in our study and others. Prenatal malnourishment leads to a decrease in glutamate binding in the adult brain [76]. Furthermore, these authors reported reduced Na^+^-independent ^3^H-glutamate binding in cellular membranes, indicating a decrease in the glutamatergic activity [76]. These finding implicate dysfunction of the glutamatergic system that may account for deficits in LTP observed in our study and others.

In addition to alterations in transmitter systems, neuroanatomical changes related to undernutrition and malnutrition may play a role in deficits observed in LTP. Prenatal protein malnutrition causes alterations in the developmental time course of dentate granule cell, leads to a significant reduction in granule cell density, reduces the complexity of dendritic spines located on granule cells apical dendrites (corresponding to the area of perforant path innervation), and alters hippocampal morphology [60], [77], [78], [79]. Additionally, undernutrition results in the permanent reduction in the number of dentate granule cell synapses [80].

Our current work suggests that prenatal undernutrition negatively influences DG synaptic plasticity in later life. We also demonstrate that the timing of caloric restriction does not influence the magnitude of the insult. The reduction in synaptic plasticity observed may underlie deficits in cognitive processes that result from prenatal undernutrition and malnutrition [81], [82], [83], [84]. Interestingly, the effects of prenatal undernutrition were only observed following TBS, suggesting that the capacity for LTP exists and can be evoked following a robust stimulation (i.e. HFS).

The differences in the pattern of results for the PF and PNEE offspring indicate that these manipulations are affecting hippocampal development in different ways. Following TBS and HFS, ethanol's effects on LTP are restricted to the second trimester, whereas deficits in PF animals (1^st^ and 2^nd^ trimester) are observed in TBS-LTP but not HFS-LTP. Therefore, a dietary deficit cannot explain all the effects of prenatal ethanol exposure, as PNEE animals would have shown a 1st trimester deficit as do the PF animals, yet do not. In the case where LTP levels in PNEE and PF animals are not significantly different, ethanol's negative effect on LTP may be the partially explained by nutritional alterations that are caused by ethanol consumption (since ethanol dams are given ad libitum access to their liquid diet). Therefore, the present findings demonstrate that PNEE is detrimental to DG development and that there is a need for refinement in the liquid diet animal model of FASD, one in which undernutrition, malnutrition, and FASD may be further teased apart.

### Summary

In summary, the hippocampal DG is vulnerable to ethanol induced alterations in long-term potentiation particularly during the 2nd trimester of pregnancy. The different effects of ethanol at different time points likely results from targeting of different developmental processes. This is not to say that ethanol does not have long-lasting effects on offspring when consumed at other time points during pregnancy, since this study only examined one plastic event in one region of the adult brain. As an example, exposure to ethanol during the 1st trimester has been shown to produce severe facial dysmorphology and interfere with organization of the brain [51], [85].

## Materials and Methods

### Animals and Breeding

Breeding animals were obtained from Charles Rivers and housed in standard cages in colony rooms kept at a constant temperature of 21°C and maintained on a 12 hour light/dark cycle. All animals were given ad libitum access to food and water, except when being administered ethanol or pair-fed diets. This study was performed in strict accordance with the guidelines established by the Canadian Council on Animal Care. The protocol was approved by the University of Victoria's Animal Care Committee (Protocol Numbers: 2010–012 and 2010–011).

Virgin female Sprague-Dawley rats were paired with breeding males in standard cages with the addition of a metal enrichment tube. Pregnancy checks were performed in the early morning via a vaginal swab and visualized on a microscope slide with an Olympus microscope (Olympus CX21, Center Valley, PA, USA) for the presence of sperm. The presence of sperm was used to indicate GD 1. During pregnancy, females were weighed on GD 1, 7, 14 and 21. The day on which females gave birth was designated as PD 1. On PD 2/3, litters were culled to 10 pups and both dams and pups were weighed on PD 8, PD 15 and PD 22. The pups generated were then weaned and group housed according to gender on PD 22/23. Due to unknown pregnancies, some maternal data is missing for control/ad libitum (AL) dams.

### Administration of the Liquid Ethanol Diets

To model ethanol exposure during an equivalent human trimester of pregnancy, rat dams or pups were exposed to a liquid diet containing ethanol either during the (Figure 1): (i) 1st trimester-equivalent  =  prenatal ethanol exposure 1 (PNEE_1_), pregnant dams given ad libitum access to the ethanol liquid diet from GD 1–11; (ii) 2^nd^ trimester-equivalent  =  prenatal ethanol exposure 2 (PNEE_2_), pregnant dams given ad libitum access to the ethanol liquid diet from GD 11–21; or (iii) 3^rd^ trimester-equivalent  =  ethanol exposure 3 (EE_3_), pups were feed an ethanol liquid diet through intubation from PD 4–9. The following administration periods and doses were chosen because of their reported effects on hippocampal development and common use in the FASD field.

PNEE dams were given ad libitum access to a liquid diet containing ethanol (35.5% ethanol derived calories; 6.61% v/v). PNEE dams were slowly introduced to the ethanol liquid diet during the first three days of administration by combining 1/3 ethanol diet with 2/3 pair-fed diet on the first day, 2/3 ethanol diet with 1/3 pair-fed diet on the second day, and 3/3 ethanol diet for the remainder of the diet administration period. Following the end of the specified diet administration period, dams were again given ad libitum access to standard rat chow.

Intubation treatments were administered three times a day from PD 4 to PD 9. For each round of intubations, pups were removed from the dam as a litter and kept on a 37°C heating pad. A premeasured length of polyethylene tubing-10 was lubricated with corn oil and gently inserted down the pup's esophagus into the stomach [19], [86]. EE_3_ pups were intubated twice daily, two hours apart, and infused with a milk/ethanol solution containing 11.9% (v/v) ethanol, totaling 5.25 g/kg of ethanol a day. This was followed by a milk only intubation, to supplement EE pups with calories that were lost due to a reduction in suckling.

Appropriate PF, SI, and AL animals were also reared. PF dams were offered an equivalent quantity of food in g/kg matching that consumed by a PNEE dam on the corresponding day of gestation. SI_3_ pups received intubations, without infusion of any solution, since milk infusions are known to abnormally accelerate the growth of SI pups [87]. AL dams and pups were given ad libitum access to standard rat chow. All groups had ad libitum access to water throughout gestation.

### Blood Ethanol Concentrations

To assess peak BECs, blood samples were obtained by way of a tail clip from pregnant dams or postnatal pups: on GD 9 from PNEE_1_ dams, GD 19 from PNEE_2_ dams, and from EE_3_ pups on PD 4. These samples were collected five hours after the presentation of the ethanol diet from PNEE dams and ninety minutes after the last milk/ethanol intubation from EE_3_ pups. Blood was centrifuged 24 hours after collection and plasma was collected and stored at −20°C until assay. BECs were analyzed using an Analox GL-5 Alcohol Analyzer (Analox Instruments, Lunenburg, MA, USA).

### Slice Preparation

Between PD 50–70, offspring were anesthetized with isoflurane, rapidly decapitated, and their brains removed in oxygenated (95% O2/5% CO2), ice-cold normal artificial cerebral spinal fluid (nACSF). nACSF contained (in mM) 125.0 NaCl, 2.5 KCl, 1.25 NaH2PO4, 25.0 NaHCO3, 2.0 CaCl2, 1.3 MgCl2, and 10.0 dextrose (pH 7.3). Transverse hippocampal slices (350 μm) were generated using a Vibratome Sectioning System 1500 (Ted Pella, Redding, CA, USA). Slices were kept in order using a modified 24-well plate and incubated in continuously oxygenated nACSF maintained at 30°C. Sections were allowed to rest for a minimum of 1 hour before recordings commenced.

### Recordings

Field recordings were collected in nACSF using an Axon MultiClamp 700B amplifier and Clampex 10.2 software (Molecular Devices, CA, USA). Using an Olympus BX51 microscope and motorized micromanipulators (Siskyou Design, OR, USA), electrodes were placed in the medial molecular layer of the DG, approximately 200 µm apart. Field excitatory postsynaptic potentials (fEPSPs) were elicited by delivering a 120 µs (10–40 µA) current pulse to the medial perforant path by way of a digital stimulus amplifier (Getting Instruments, CA, USA) and a single concentric bipolar stimulating electrode (FHC, Bowdoin, ME, USA). fEPSPs were recorded using a single glass recording electrode (0.5–1.5 MΩ) filled with nACSF. A modified I/O experiment was conducted in which the stimulation magnitude was increased until a maximal response prior to population spike appearance was acquired. Stimulation magnitude was then set to elicit approximately 50% of the maximal response. A PP experiment was conducted using an inter-pulse interval of 50 ms (5x; 15 s between pairings).

Baseline measurements were collected using fEPSPs evoked every 15 seconds. A stable baseline of 20 minutes was required before a conditioning stimulus could be applied to the slice. Baseline stimulation parameters were returned to immediately following the conclusion of the conditioning stimulus and fEPSPs were recorded for a minimum of 60 minutes. An I/O experiment was then conducted with increasing stimulation magnitude (30 to 300 µs pulse width; 15 s intervals).

### Conditioning Stimulus Protocols

LTP of fEPSPs was induced using one of two conditioning stimuli: (i) HFS or (ii) TBS. HFS consisted of four trains of 50 pulses at 100 Hz, 30 seconds apart; whereas, TBS consisted of four pulses at 100 Hz followed 200 ms later by another burst of four pulses, occurring five times with a 30 second inter-train interval. The GABA_A_ receptor antagonist bicuculline methiodide (Sigma-Aldrich, Oakville, ON, Canada) was included in the nACSF during baseline and CS recordings for all experiments. Bicuculline methiodide was prepared as a concentrated stock solution and diluted with nACSF to a concentration of 10 μM prior to each recording.

### Data and Statistical Analysis

All electrophysiological data analysis was conducted with Axon ClampFit 10.2 software (Molecular Devices, CA, USA). The initial slope of the fEPSP was measured and used for all data analysis. PP ratios were calculated by dividing the slope of the second fEPSP by the first and converting this number into a percent change. I/O curves were calculated by normalizing recordings to the value of the 5^th^ pulse and reported as a percent change, with the 5^th^ pulse equaling 100%. For all other experiments, recordings were normalized to the average value of the 20 minute baseline and reported as percent change from baseline. LTP was calculated by averaging the last 20 traces (i.e., 55–60 min) of the post-CS recording.

Overall effects across trimester equivalents were examined with a two-way factorial analysis of variance (ANOVA): developmental treatment (AL, PNEE_1_, PF_1_, PNEE_2_, PF_2_, EE_3_, SI_3_) x gender (male, female). In the case of I/O analysis, a repeated measures ANOVA was conducted. Significant main effects and interactions were further analyzed with Tukey-HSD post hoc tests. T-tests were also used to compare between groups, when appropriate. For all studies, data was presented as means ± standard error of the mean (SEM). For graphing purposes, data from both male and female offspring were pooled together since no main effect of gender was obtained. Results were processed for statistical analysis using Statistica 7.0 (Statsoft, Inc., Tulsa, OK, USA) and differences were considered significant when p<0.05.
